# Supplementary material for: Hydrological and topographic determinants of biomass and species richness in a Mediterranean-climate shrubland
Source: PLoS One. 2021 May 27;16(5):e0252154. doi: 10.1371/journal.pone.0252154 (PMC8158923; doi:10.1371/journal.pone.0252154)
Supplement: S2 Table — NA = not available, the date that could not be encountered, or species that were seen outside the plot. (DOCX) [file pone.0252154.s004.docx]

Supporting Information to the paper Díaz de León, Méndez-Alonzo, Bullock, and Vivoni. Hydrological and topographic determinants of biomass and species richness in a Mediterranean-climate shrubland

**S2 Table.** Table of species abbreviation (Abb) list and their contribution with relative cover, life form, life cycle, height in meters (H), relative stem mass, relative leaf area, frequency and plant habit. NA= not available, the date that could not be encountered, or species that were seen outside the plot.

| **Species** | **Abb** | **Family** | **Authority** | **Relative**  **cover (%)** | **Life form** | **Life cycle** | **H** | **Relative stem mass (%)** | **Relative leaf area (%)** | **Frequency** | **Plant Habit** |
| --- | --- | --- | --- | --- | --- | --- | --- | --- | --- | --- | --- |
| *O****r****nithostaphylos oppositifolia* | Oo | Ericaceae | (Parry) Small. | 30.8 | Shrub | Perennial | 2.2 | 9.8 | 26.4 | 13 | Spreading |
| *Eriogonum fasciculatum* | Ef | Polygonaceae | Benth. | 17.52 | Shrub | Perennial | 1.1 | 19.7 | 14.5 | 19 | Spreading |
| *Adenostoma fasciculatum* | Af | Rosaceae | Hook & Arn. | 16.7 | Shrub | Perennial | 1.8 | 21.7 | 11.9 | 12 | Spreading |
| *Xylococcus bicolor* | Xb | Ericaceae | Nutt. | 6.4 | Shrub | Perennial | 1.6 | 5.7 | 3 | 3 | Spreading |
| *Rhamnus crocea* | Rc | Rhamnaceae | Nutt. | 3.94 | Shrub | Perennial | 1.6 | 0.06 | 0.07 | 4 | Spreading |
| *Quercus berberidifolia* | Qb | Fagaceae | Liebmann | 3.93 | Shrub | Perennial | 2.1 | 9.7 | 4.5 | 1 | Spreading |
| *Cneoridium dumosum* | Cd | Rutaceae | (Nutt. ex Torr. & Gray) Baill. | 3.53 | Shrub | Perennial | 1.3 | 5.7 | 7.6 | 9 | Spreading |
| *Salvia apiana* | Sa | Lamiaceae | Jeps. | 3 | Shrub | Perennial | 1.0 | 0.2 | 1 | 4 | Spreading |
| *Malosma laurina* | Ml | Anacardiaceae | (Nutt.) Nutt. ex Abrams | 2.6 | Shrub | Perennial | 2.2 | 29.2 | 22 | 6 | Spreading |
| *Gutierrezia sarothrae* | Gs | Asteraceae | (Pursh) Britton & Rusby | 2.1 | Shrub | Perennial | 0.4 | 0.1 | 1 | 12 | Spreading |
| *Romneya*  *trichocalyx* | Rt | Papaveraceae | Eastw. | 1.7 | Shrub | Perennial | 2.9 | NA | NA | 2 | Spreading |
| *Fraxinus parryi* | Fp | Oleaceae | Moran | 1.5 | Shrub | Perennial | 3.6 | 1.3 | 2.6 | 3 | Erect |
| *Artemisia california* | Ac | Asteraceae | Less. | 1.5 | Shrub | Perennial | 1.1 | 0.3 | 2.1 | 2 | Spreading |
| *Keckiella antirrhinoides* | Ka | Plantaginaceae | (Benth.) Straw | 1.4 | Shrub | Perennial | 1.2 | 0.06 | 0.07 | 5 | Spreading |
| *Eriodictyon sessilifolium* | Es | Boraginaceae | Greene | 1.1 | Shrub | Perennial | 2.2 | 0.1 | 0.3 | 3 | Spreading |
| *Trichostema parishii* | Tp | Lamiaceae | Vasey | 0.5 | Shrub | Perennial | 0.5 | NA | NA | 2 | Spreading |
| *Acmispon glaber* | Ag | Fabaceae | (Vogel) Brouillet | 0.4 | Shrub | Perennial | 0.7 | 0.1 | 0.5 | 7 | Spreading |
| *Crocanthemum scoparium* | Cs | Cistaceae | (Nutt.) Millsp. | 0.4 | Herbs | Perennial | 0.5 | 0.4 | 2.4 | 5 | Spreading |
| *Hazardia squarrosa* | Hs | Asteraceae | (Hook. & Arn.) Greene | 0.3 | Herbs | Perennial | 0.6 | 0.03 | 0.3 | 5 | Erect |
| *Ceanothus greggii* | Cg | Rhamnaceae | A. Gray | 0.2 | Shrub | Perennial | 1.6 | 0.02 | 0.1 | 1 | Spreading |
| *Acourtia microcephala* | Am | Asteraceae | DC. | 0.04 | Herbs | Perennial | 0.3 | NA | NA | 1 | Spreading |
| *Chlorogalum*  *parviflorum* | Cp | Agavaceae | S. Watson | 0.01 | Herbs | Perennial | 0.03 | NA | NA | 3 | Prostrate |
| *Bahiopsis laciniata* | Bl | Asteraceae | (A. Gray) E: E. Shilling & Panero | <0.01 | Shrub | Perennial | NA | NA | NA | NA | Spreading |
| *Encelia californica* | Ec | Asteraceae | Nutt. | <0.01 | Shrub | Perennial | NA | NA | NA | NA | Spreading |
| *Cuscuta californica* | Cc | Convolculaceae | Hook. & Arn. | <0.01 | Herbs | Annual | NA | NA | NA | NA | Parasitic |
| *Hesperoyucca whipplei* | Hw | Asparagaceae | (Torr.) Trel. | <0.01 | Rosette | Perennial monocarpic | NA | NA | NA | NA | Spreading rosette |
| *Marah macrocarpa* | Mm | Cucurbitaceae | Greene | <0.01 | Herbs | Perennial | NA | NA | NA | NA | Climbing |
| *Tetradymia comosa* | Tc | Asteraceae | A. Gray | <0.0001 | Herbs | Perennial | 0.3 | NA | NA | NA | Spreading |
| *Xanthisma junceum* | Xj | Asteraceae | (Greene) D. R. Morgan & R. L. Hartm | <0.0001 | Herbs | Perennial | NA | NA | NA | NA | Scandent |
