# Supplementary figures and images for: Hydrological and topographic determinants of biomass and species richness in a Mediterranean-climate shrubland
Source: PLoS One. 2021 May 27;16(5):e0252154. doi: 10.1371/journal.pone.0252154 (PMC8158923; doi:10.1371/journal.pone.0252154)

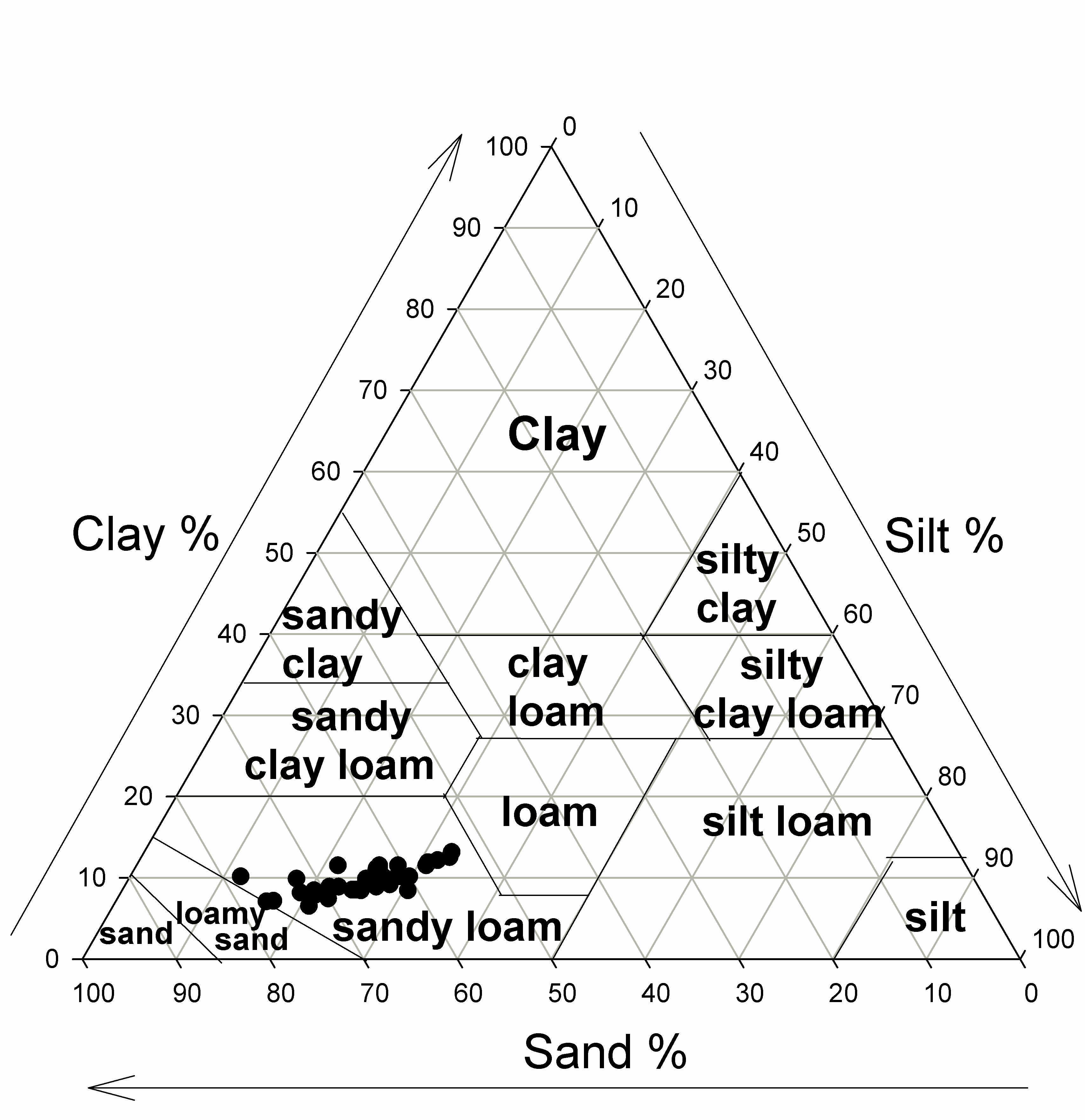

Supplement: S1 Fig — (TIF) [file pone.0252154.s010.tif]

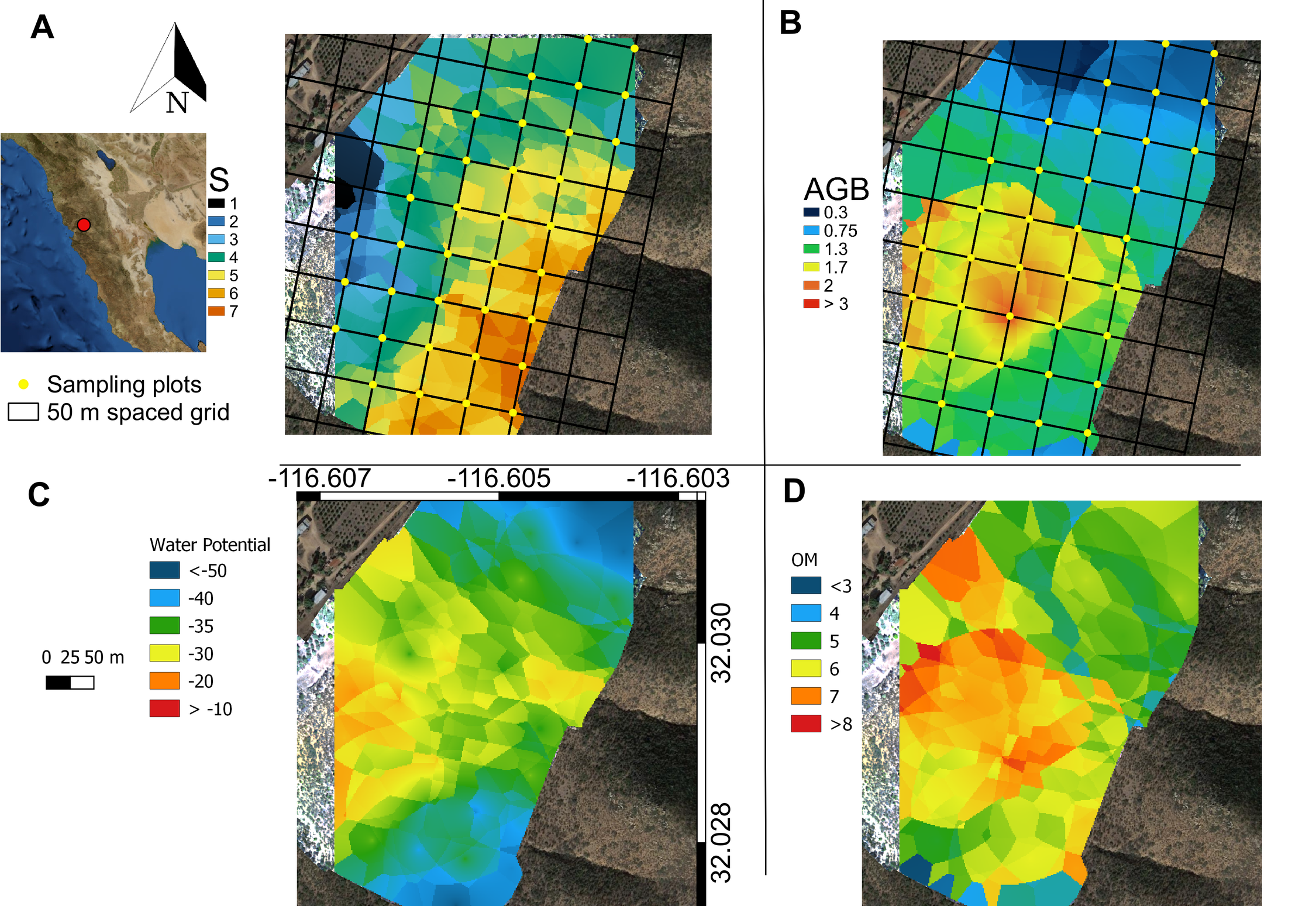

Supplement: S2 Fig — (TIF) [file pone.0252154.s011.tif]

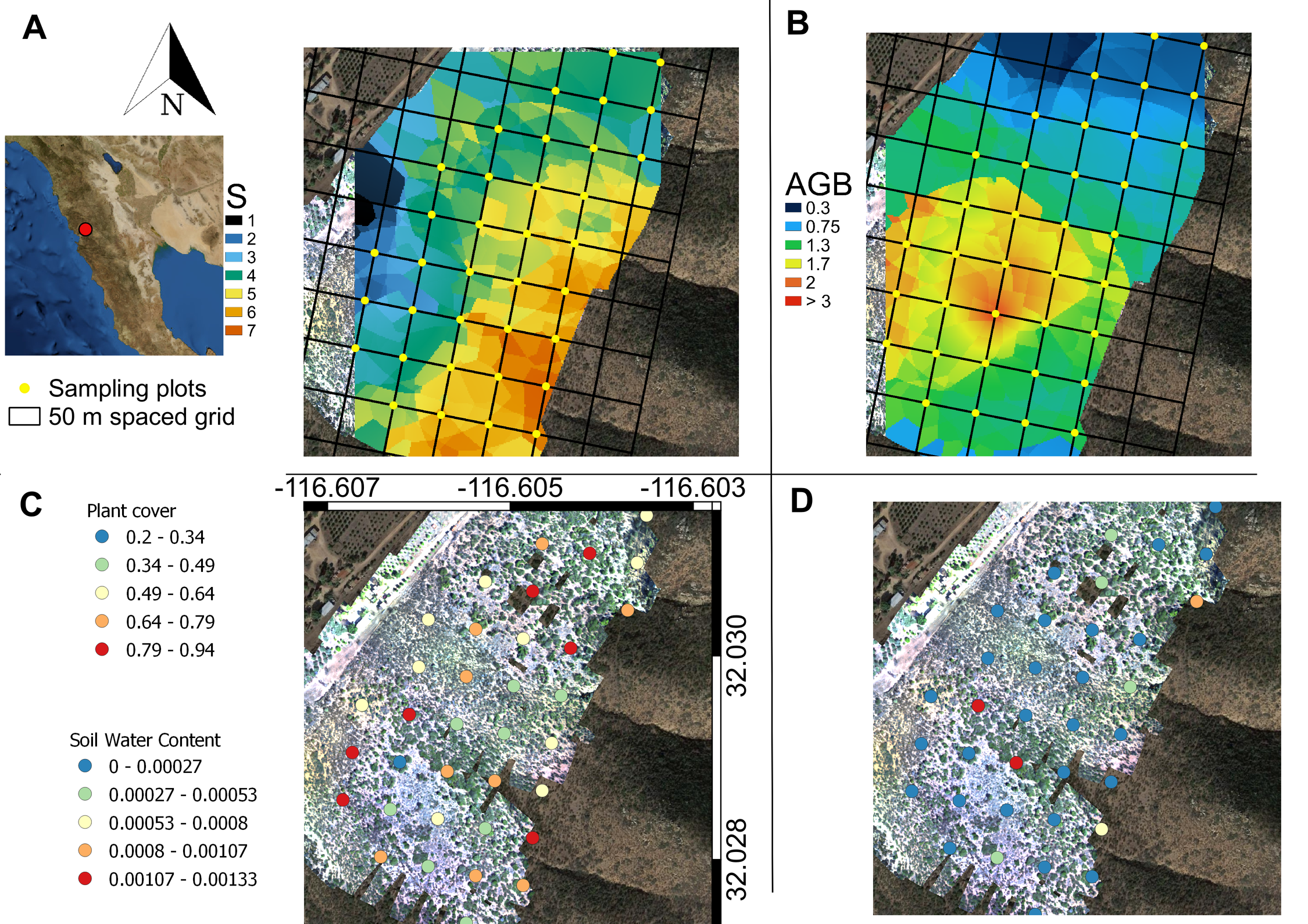

Supplement: S3 Fig — (TIF) [file pone.0252154.s012.tif]
