# Supplementary material for: Hydrological and topographic determinants of biomass and species richness in a Mediterranean-climate shrubland
Source: PLoS One. 2021 May 27;16(5):e0252154. doi: 10.1371/journal.pone.0252154 (PMC8158923; doi:10.1371/journal.pone.0252154)
Supplement: Attachment — Submitted filename: reviewer_comments_MS_PONE-D-20-36492.docx [file pone.0252154.s013.docx]

The authors studied a chaparral ecosystem to understand drivers of aboveground biomass and plant species richness, and their relationship. The authors found a positive relationship between aboveground biomass and water availability, while species richness covaried with landscape properties and reduced shrub cover. These are interesting findings, although the positive effect of increased water availability on biomass is no surprise, as well as the evidence for spatial heterogeneity of physical environment increasing the importance of species richness. I further suggest in my comments, some analyses and illustrations that would enhance the scope of the paper.

*Title*

Lines 5-6: Should title instead be something like “Determinants of biomass and species richness in a Mediterranean-climate shrubland” since species richness best correlated with other variables?

*Introduction*

Line 62-63: state clearly that water availability is one of the main factors influencing plant species richness. Other factors, such as community assembly factors (e.g., seed propagule) or disturbance, could also influence plant species richness.

Lines 64-65: The connection between aboveground net productivity and precipitation to plant species richness is not clear in this sentence. Perhaps authors meant to say something like “Although, plant species richness has been related to several ecosystem functions in drylands, association to productivity remains unclear”.

Lines 75-76: What does “Positive-reinforcement processes” mean in this context?

Line 93-95: including facilitative relationships in some cases. See

Species diversity enhances ecosystem functioning through interspecific facilitation

BJ Cardinale, MA Palmer, SL Collins

Nature 415 (6870), 426-429

Lines 103-104: replace “tests” for “studies” and add “in this system” after abiotic factors. Also, authors mention that the relationship between AGB and *S* is not fully understood, but what is known in this system?

Line 118: add reference that supports hypothesis

Lines 118-122: and what is the hypothesis for these other variables with the relationship between *S*-ABG, and for each (*S* and ABG)?

Line 222: I didn’t see variable scaling in the authors code (see S2 Appendix stepwise analysis Data plot.txt), considering that variables had different units. Does variables scaling influence the outcome of the results?

Lines 122-123: how will generated maps of AGB and *S* by interpolation techniques support statistical analysis in this study?

*Methods*

Line 127: In figure 1, authors refer to 36 sampling plots and later in line 158 first mention them in the methods, however, it is unclear why 36 plots were chosen.

Lines 132-134: Because this system has such marked seasons, can authors provide means for precipitation broken down by dry and wet seasons within the same time range (1986-2018)? Time range should include the period of observations.

Line 138: in “has been sporadically used by cattle”, did cattle have access to the plots for grazing? If so, it should be acknowledged that study plots were eventually grazed since this can influence the outcome of plant richness and biomass.

Lines 138-139: clarify why this information is relevant.

Line 146: why was initial mapping done and how often? Was it for site characterization in table 1? If so, say this up front in this paragraph.

Lines 161: Explain why only one observation was performed. Is there any previous knowledge of the system (e.g., no change in species composition within and across seasons) that would justify one observation in April?

Lines 162-164: I am not familiar with this technique to assess plant cover. How precise were authors in determining plant cover by using images obtained 5m from the ground? Especially for short plants and considering various layers of the vegetation. If available, provide citations for the use of this technique and explain how species-specific cover was quantified from images.

Line 168: nomenclature for what? Clarify this in the text.

Line 170: clarify why AGB was harvested in February 2018 and in how many replicates. Was February the peak of biomass for this system?

Line 173-174: clarify what authors meant by “species origin of the remains”. Was it dead biomass?

Lines 178-182: Do the authors have any information about how abundant (average cover) these groups species are and if their absence in the harvested plots would influence sampled AGB values?

Lines 206-208: how did authors get to the best estimator of plant richness?

*Results*

It would be very helpful and it would add to the paper if authors could add other layers to the map in Figs 1C and 1D for the distribution of key soil properties and shrub cover – i.e. key variables driving AGB and *S*. For example, mark areas in the map where soil water content was high and low.

Line 310: there’s only mention to dry fruit mass, were all or most of the species in this reproductive stage? If so, how much information in terms of biomass and greenness was potentially missed if vegetation sampling was done past flowering? I would expect peak of greenness or productivity to occur prior reproductive events. This would be related to resource accumulation for investment in reproductive structure. Or does this does not apply to this system? Please clarify.

*Discussion*

Lines 453:355: not sure if authors are trying to say this, but reduced species number could also be part of species phenology, meaning that the timing of activity occurs in the wet season and plants senesce before the dry season. This makes me wonder if ‘filtered species’ could have contributed significantly to the species number during measurements.

Lines 371-372: is there any influence of cattle grazing here? Grazing normally increases species richness. If so, how grazed is this study site?

Lines 434 and line 443: a suggestion to better explore AGB-*S* relationship: because unequal relative abundance is so marked in this study site, authors would benefit from using Community Weighted Means (CWMs) at the plot-level. This analysis would weight the relative abundance of species into AGB and then authors could test the CWM AGB - *S* relationship. See package *FD*, function *functcomp* in R.

Line 452: what type of disturbance? Grazing?

*Supporting information*

S1 Table species abbreviation – add a column for species life cycle (perennial, annual or biannual)
